# Structural differences among children, adolescents, and adults with attention-deficit/hyperactivity disorder and abnormal Granger causality of the right pallidum and whole-brain

**DOI:** 10.3389/fnhum.2023.1076873

**Published:** 2023-02-14

**Authors:** Elijah Agoalikum, Benjamin Klugah-Brown, Hongzhou Wu, Peng Hu, Junlin Jing, Bharat Biswal

**Affiliations:** ^1^The Clinical Hospital of Chengdu Brain Science Institute, MOE Key Laboratory for Neuroinformation, School of Life Science and Technology, University of Electronic Science and Technology of China, Chengdu, Sichuan, China; ^2^Department of Biomedical Engineering, New Jersey Institute of Technology, Newark, NJ, United States

**Keywords:** attention deficit/hyperactivity disorder, age, voxel-based morphometry, Granger causality analysis, resting-state fMRI

## Abstract

Attention-deficit/hyperactivity disorder (ADHD) is a childhood mental health disorder that often persists to adulthood and is characterized by inattentive, hyperactive, or impulsive behaviors. This study investigated structural and effective connectivity differences through voxel-based morphometry (VBM) and Granger causality analysis (GCA) across child, adolescent, and adult ADHD patients. Structural and functional MRI data consisting of 35 children (8.64 ± 0.81 years), 40 adolescents (14.11 ± 1.83 years), and 39 adults (31.59 ± 10.13 years) was obtained from New York University Child Study Center for the ADHD-200 and UCLA dataset. Structural differences in the bilateral pallidum, bilateral thalamus, bilateral insula, superior temporal cortex, and the right cerebellum were observed among the three ADHD groups. The right pallidum was positively correlated with disease severity. The right pallidum as a seed precedes and granger causes the right middle occipital cortex, bilateral fusiform, left postcentral gyrus, left paracentral lobule, left amygdala, and right cerebellum. Also, the anterior cingulate cortex, prefrontal cortex, left cerebellum, left putamen, left caudate, bilateral superior temporal pole, middle cingulate cortex, right precentral gyrus, and the left supplementary motor area demonstrated causal effects on the seed region. In general, this study showed the structural differences and the effective connectivity of the right pallidum amongst the three ADHD age groups. Our work also highlights the evidence of the frontal-striatal-cerebellar circuits in ADHD and provides new insights into the effective connectivity of the right pallidum and the pathophysiology of ADHD. Our results further demonstrated that GCA could effectively explore the interregional causal relationship between abnormal regions in ADHD.

## 1 Introduction

Attention-deficit/hyperactivity disorder (ADHD) is a mental health disorder characterized by inattentive, hyperactive, or impulsive behaviors (American Psychiatry Association, 2000). ADHD is a childhood disorder but often persists to adulthood (Danielson et al., 2018). According to the Diagnostic and Statistical Manual of Mental Disorders-IV (DSM-IV), ADHD is divided into three (3) sub-types, which are; hyperactive/impulsive (HI), inattentive (IA), and combined (C) type. The prevalence of ADHD in children, adolescents, and adults is said to be 9.5%, 11.4%, and 4.4%, respectively (Gimpel and Kuhn, 2000; Barbaresi et al., 2002; Kessler et al., 2006; Wolraich et al., 2011), with symptoms varying across different age groups (Katragadda and Schubiner, 2007). There has been accumulated pathophysiological evidence from structural and functional neuroimaging studies of ADHD (Seidman et al., 2005; Frodl and Skokauskas, 2012; Agoalikum et al., 2021).

In recent times, voxel-based morphometry (VBM) has been employed to study structural deficits of the brain in several neurodegenerative disorders on a voxel-by-voxel or seed-to-voxel scale. Structural magnetic resonance imaging (sMRI) studies using VBM and meta-analysis has shown that some brain regions in ADHD patients exhibit reduced volumes relative to age-matched healthy control subjects. Using cross-sectional mega-analysis, Hoogman and colleagues reported reduced volumes in the accumbens, caudate, putamen, amygdala, and hippocampus in ADHD patients compared to healthy controls (Hoogman et al., 2017). Furthermore, a meta-analysis study combining morphometry and manual voxel tracking showed that, in ADHD children, the basal ganglia structure, such as the right globus pallidus, right putamen, and caudate nucleus, is profoundly influenced. In contrast, the cerebrum regions of adult patients primarily change in the marginal areas, such as the ACC and amygdala (Frodl and Skokauskas, 2012). The basal ganglia and linked regions consist of various subcortical cell groups, which perform a range of roles, such as motor control, motor learning, executive function and behavior, and emotional control (Mello and Villares, 1997). Also, the abnormalities in the basal ganglia are consistent with previous models of frontostriatal pathway dysfunction, which may be associated with the dopaminergic and norepinephrine systems imbalance observed in ADHD (Frodl, 2010). The dysfunction in these altered brain regions coincides with the clinical manifestations of children with ADHD, which provides a good direction for future clinical treatment. Also, the limbic system is a circular area on the medial side of the brain, consisting of multiple parts associated with the limbic networks of many mental symptoms, which participate in emotion, behavior, motivation, self-protection, sexual behavior, social activity, and memory. The functional impairment of the marginal division could explain the material premise of the transformation of ADHD into depression, mania, and other mental disorders, but the specific transformation mechanism from childhood to adulthood remains inadequately studied.

To examine the relationships between altered gray matter regions and the whole-brain or set of defined regions, Granger causality analysis (GCA) has been widely employed in neuroimaging studies (Rypma et al., 2006; Zang et al., 2012). This method is a statistical technique used to depict information flow by determining whether one time series precedes and allows the prediction of another time series. Thus, the neural activity in one brain region precedes and enables the prediction of the neural activity in another. GCA has been used in several neurodegenerative disorders, including; frontal lobe epilepsy (Klugah-Brown et al., 2019), and ADHD (Lan et al., 2021), among others.

Even though VBM has been used to determine gray matter volume differences between ADHD patients and healthy control subjects, no study has employed this method to investigate structural differences across three different age groups of ADHD patients. In view of this, we employed VBM first to investigate the structural differences across child, adolescent, and adult ADHD subjects, and GCA to explore the effective connectivity between the right pallidum and the rest of the brain and the directional causal relationships among ROIs.

## 2 Materials and methods

### 2.1 Data acquisition

Resting-state functional and structural MRI data of ADHD patients (158 subjects) were obtained from the New York University Child Study Center for the ADHD-200 Global Competition and UCLA dataset (Bilder et al., 2016). The NYU dataset comprises 45 child ADHD patients and 73 adolescent ADHD patients, and the UCLA dataset is made up of 40 adult ADHD patients. Both datasets are made open to researchers online. For child and adolescent groups, psychiatric diagnoses were based on evaluations with the Schedule of Affective Disorders and Schizophrenia for Children—Present and Lifetime Version (KSADS-PL) administered to parents and children and the Conners’ Parent Rating Scale-Revised, Long version (CPRS-LV). Intelligence was evaluated with the Wechsler Abbreviated Scale of Intelligence (WASI). Inclusion in the ADHD group required a diagnosis of ADHD based on parent and child responses to the KSADS-PL as well as on a T-score greater than or equal to 65 on at least one ADHD related index of the CPRS-R: LV. Psychostimulant drugs were withheld at least 24 h before scanning. Estimates of FSIQ above 80, right-handedness, and absence of other chronic medical conditions were required for all participants”. For the adult group, participants were men or women ages 21–50 years; NIH racial/ethnic category either White, not Hispanic or Latino; or Hispanic or Latino, of any racial group; primary language either English or Spanish; completed at least 8 years of formal education; no significant medical illness; adequate cooperation to complete assessments; visual acuity 20/60 or better; and urinalysis negative for drugs of abuse (Cocaine; Methamphetamine; Morphine; THC; and Benzodiazepines). ADHD criteria were assessed using Adult ADHD Interview and excluded anyone with other disorders; stable medications were permitted for patients. For MRI studies we excluded participants who were left handed, who believed they might be pregnant or had other contraindications to scanning (e.g., claustrophobia, metal in the body, body too large to fit in the scanner). Diagnoses followed the DSM-IV—Text Revision and were based on the Structured Clinical Interview for DSM-IV (SCID-I) supplemented by the Adult ADHD Interview (a structured interview form derived from the KSADS-PL, in order to enable a more detailed characterization of lifetime history of ADHD in adults. Interviewers/raters were trained according to the criteria; in brief minimum standards of acceptable symptom agreement were overall kappa of 0.75, a kappa specificity of 0.75, and sensitivity of 0.75, and 0.85 kappa for diagnostic accuracy. Diagnostic and Symptom elicitation skill was also assessed with the SCID Checklist of Interviewer Behaviors and the Symptom Checklist of Interviewer Behaviors. Ongoing quality assurance checks documented kappa above 0.75 for each rater annually during the course of the study. All participants used in the current study were diagnosed with ADHD, and their scores have been used for the correlation analysis in the present study. Detailed information about the subjects can be found in Table 1.

**Table 1:** Data demographics.

ADHD group	Adults (*n* = 39)	Adolescents (*n* = 40)	Children (*n* = 35)	*P*-value
Gender (M/F)	(20/19)	(31/9)	(26/9)	= 0.0262
Age (years)	31.59 ± 10.13	14.11 ± 1.83	8.64 ± 0.81	<0.00001
Data Range (years)	21–50	11.41–17.61	7.24–9.98	
OA Score	63.49 ± 4.99	70.18 ± 46	70.74 ± 7.81	
HI Score	31.57 ± 4.63	65.95 ± 11.89	66.69 ± 12.69	
IA Score	35.77 ± 2.78	68.88 ± 9.16	69.89 ± 8.87	

One-ANOVA was used to determine group differences. OA, Overall Disease severity; HI, Hyperactivity severity; IA, Inattentive severity.

For the adult dataset, MRI data were acquired on one of two 3T Siemens Trio scanners, located at the Ahmanson-Lovelace Brain Mapping Center (Siemens version syngo MR B15) and the Staglin Center for Cognitive Neuroscience (Siemens version syngo MR B17) at UCLA. Functional MRI data were collected using a T2*-weighted echo-planar imaging (EPI) sequence with the following parameters: slice thickness = 4 mm, slices = 34, TR = 2 s, TE = 30 ms, flip angle = 90°, matrix 64 × 64, FOV = 192 mm. A T2-weighted matched-bandwidth high-resolution anatomical scan (with the same slice prescription as the fMRI scan) and MPRAGE were collected. The parameters for the high-resolution scan were: 4 mm slices, TR/TE = 5,000/34 ms, 4 averages, matrix = 128 × 128, 90-degree flip angle. The parameters for MPRAGE were the following: TR = 1.9 s, TE = 2.26 ms, FOV = 250 mm, matrix = 256 × 256, sagittal plane, slice thickness = 1 mm, 176 slices. For the child and adolescent datasets, MRI data were obtained using Siemens Magnetom Allegra Syngo Mr 2004a. FMRI data were collected using an echo-planar imaging sequence with the following parameters: slice thickness: 4 mm, Slices: 33, TR: 2,000 ms, TE: 15 ms, Rotation = 90°, FoV phase: 80.0%, FoV read = 240 mm. In addition, T1-weighted images were acquired using the following parameters: Slice thickness = 1.33 ms, TR = 2,530 ms, TE = 3.25 ms, rotation = 0 degrees, FoV phase = 100.0%, FoV read = 256 mm.

### 2.2 Data preprocessing

#### 2.2.1 Functional data preprocessing

For the adult dataset, the first two (2) time points were removed, leaving final time points of 150. For the pediatric (adolescents and children) dataset, the first 26 time points were removed to ensure that all the data have equal time points since the time courses are used in the connectivity calculations. The same preprocessing steps were done for all subjects, including slice time correction, realignment, co-registration of  T1 images to corresponding functional images, segmentation, normalization by Diffeomorphic Anatomical Registration using Exponentiated Lie algebra (DARTEL; Ashburner, 2007), and resampling to 3 × 3 × 3 mm^3^ voxels, nuisance covariates regression using Friston 24 (Friston et al., 1996), spatial smoothing with a 6 mm full width half maximum (FWHM) Gaussian kernel, linear detrending, and filtering using a bandpass filter of 0.01–0.08 Hz. Pediatric and adult datasets were preprocessed separately to ensure that the correct templates were generated for normalization. Subjects with a maximum translation >2 mm or rotation >2^o^ were excluded from further analysis, leaving 114 subjects. The final data used for further analysis included 35 children (8.64 ± 0.81 years), 40 adolescents (14.11 ± 1.83 years), and 39 adults (31.59 ± 10.13 years). All preprocessing steps were performed using the data processing assistant for resting-state fMRI, advanced edition (DPARSFA) in the DPABI toolbox (Yan et al., 2016).

#### 2.2.2 Structural data preprocessing

High-spatial-resolution T1-weighted MR imaging data were processed using the Computational Anatomy Toolbox (CAT12^1^) in statistical parametric mapping software (SPM12^2^). All T1 images were first checked for artifacts and reoriented to adjust image origins at the anterior commissure. Secondly, the images were normalized to age-specific templates created using the Template-O-Matic toolbox (TOM^3^). Segmentation was then done to separate the T1 images into gray matter (GM), white matter, and cerebrospinal fluid and resampled to a volume image resolution of 3 × 3 × 3 mm^3^. After the data quality and sample homogeneity check, the segmented GM images were smoothed using an 8-mm full width at half maximum Gaussian kernel. The smoothed GM images were used for subsequent analyses.

### 2.3 Voxel-based morphometric analysis (VBM)

To determine the gray matter alterations in ADHD, One-way Anova as implemented in SPM12 was performed on the smoothed GM images of the three ADHD age groups. Age, gender, and total intracranial volume (TIV) were used as covariates for statistical analyses. Furthermore, given that our data were obtained from different sites, and several studies have shown the effect of multi-site in different ADHD age groups (Hong et al., 2017; Zhou et al., 2019), we regressed the effects of the site in our analyses.

### 2.4 Statistical analysis

As mentioned in section 2.3 above, One-way Anova computed as implemented in SPM12; in brief, the smoothed gray matter images of all three ADHD age groups were loaded onto the SPM12 toolbox using the One-way Anova option. Gender, age, TIV and sites were used as covariates, and Family-wise error rate (FWE) correction was employed for multiple comparison corrections. In addition, we examined the relationship between altered regions and symptomatic values; we performed correlation analysis between regions that show significant gray matter differences and disease severity. To this end, we defined a sphere ROI (radius = 7 mm) using MarsBaR toolbox^4^. We then applied the general linear model (GLM) across all subjects, and using the extracted ROIs; parametric values were obtained. These values were subsequently used as independent variables against the disease severity using MATLAB partial correlation option. Age, gender, and site were regressed as covariates. Two subjects did not have disease severity scores and were excluded from the correlations analysis. FWE was used for multiple comparison corrections for all statistical analyses.

### 2.5 Granger causality analysis (GCA)

We use the signed path coefficient-based GCA implemented in the REST toolkit^5^. GCA was performed on a voxel-wise basis for all the voxels. The right pallidum region with Montreal Neurologic Institute coordinates (18, 2, -2 with radius = 7 mm) was selected from a one-way Anova test of a whole-brain voxel-based morphometric comparison among all three (3) ADHD patient groups and correlated with disease severity in the current study. We, therefore, selected this region for the GCA analysis. The order was set to one (1), and GCA was Z-transformed. GCA from the seed region (right pallidum) to the whole-brain (Outflow) and from the whole-brain to the seed region (Inflow) were measured. We further investigated the causal effect among significant whole-brain areas identified in GCA by calculating an ROI-ROI causality for outflow and inflow. The signed-path coefficient GCA was performed to build an ROI causal network that characterized the causal relationships among the ROIs. Age, gender, site, and mean framewise displacement (mean FD) were regressed out as covariates during the analyses. FWE (*P* < 0.05) was used for multiple comparison corrections.

## 3 Results

### 3.1 VBM results

**Figure 1:**
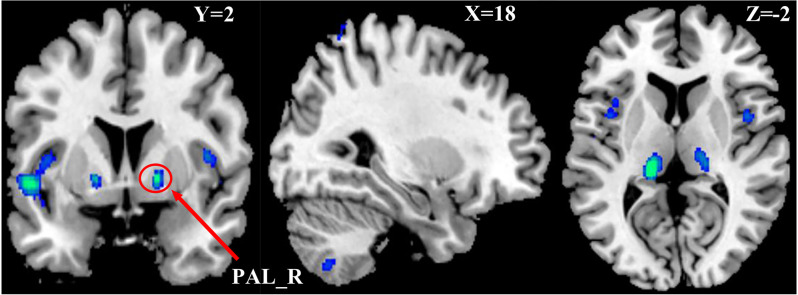
Differences in Gray Matter Volume among the three ADHD patient groups. *P* < 0.05, FWE corrected. Differences were mainly found in the basal ganglia, cerebellum, insula, and left part of the superior temporal cortex.

**Figure 2:**
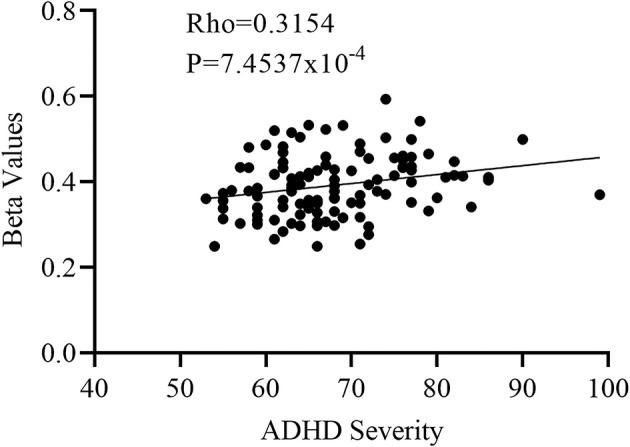
Partial correlation between the right pallidum and overall disease severity with the right pallidum showing a positive relationship with overall disease severity.

One-way Anova was used to determine the significant gray matter volume differences among the three ADHD age groups. Regions showing significant differences in gray matter volume include: the bilateral pallidum, bilateral thalamus, bilateral insula, Temporal_Sup_L, and the right cerebellum (*P* < 0.05, FWE corrected), as demonstrated in Figure 1 and Supplementary Table S1. For the correlation analysis between significant gray matter volume regions and disease severity, only the right pallidum was found to show a significant relationship (positive) with disease severity (Figure 2).

### 3.2 GCA results

**Figure 3:**
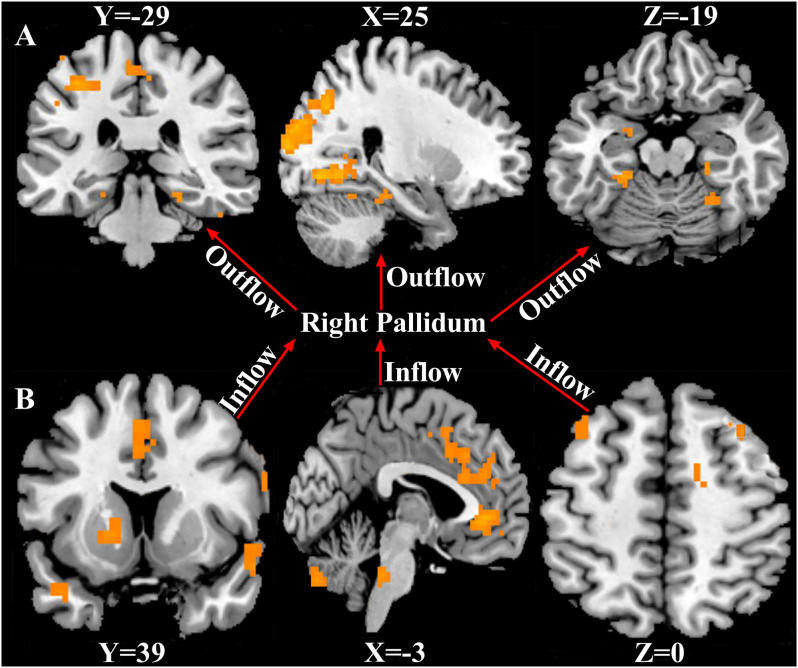
Causal connectivity to and fro the right pallidum. **(A)** Causal effect from the right pallidum to significant whole-brain regions (Outflow). **(B)** Causal effect from the significant whole-brain regions to the right pallidum (Inflow). *P* < 0.05, FWE corrected. The arrows signify the direction of causality.

Using the right pallidum as seed, significant causal effect from the seed region to the occipital_Mid_R, bilateral fusiform, postcentral_L, Paracentral_Lobule_Left, Amygdala_L, and the right cerebellum was observed (*P* < 0.05, FWE corrected). The results are shown in Figure 3A and Supplementary Table S2. Also, from Figure 3B and Supplementary Table S3, significant causal effects from the anterior cingulate cortex, cerebellum_Crus2_L, putamen_L, caudate_L, bilateral superior temporal pole, middle cingulate cortex, prefrontal cortex, precentral_R, and the Supp_Motor_Area_L to the seed region (right pallidum) was observed (*P* < 0.05, FWE corrected).

**Figure 4:**
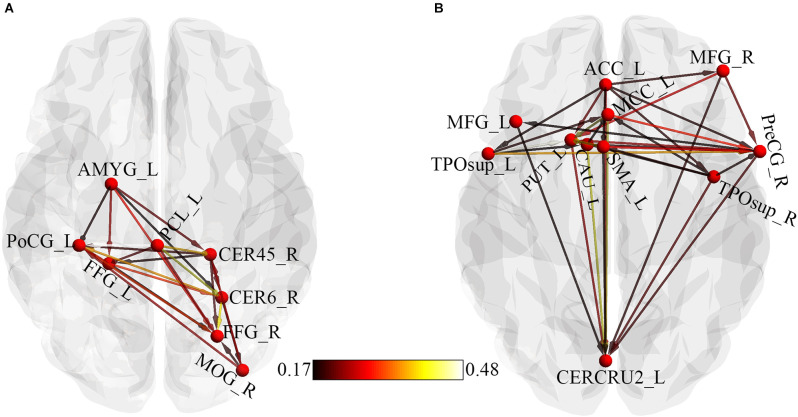
ROI-Wise GCA showing causal relationships among ROIs. **(A)** Causal relationships among outflow ROIs. **(B)** Causal relationships among inflow ROIs. The color bar indicates the connectivity strength. AMYG, Amygdala; PoCG, Postcentral gyrus; PCL, Paracentral lobule; CER45, Cerebellum_4_5; CER6, Cerebellum_6; FFG, Fusiform gyrus; MOG, Middle occipital gyrus; L, Left; ACC, Anterior cingulate cortex; MCC, Middle cingulate cortex; MFG, Middle frontal gyrus; TPOsup, Temporal pole: superior temporal gyrus; R, Right; PreCG, Precentral gyrus; SMA, Supplementary motor area; CAU, Caudate; PUT, Putamen; CERCRU2, Cerebellum_Crus2.

Regions that showed significant causality from the seed to significant whole-brain regions and from whole-brain regions to the seed were extracted and used for the ROI-ROI GCA. Figure 4 represents the results from the ROI-ROI GC analysis. The results showed directional networks revealing interregional causal relationships among the ROIs. For outflow ROI-ROI GC analysis (Figure 4A), the amygdala, postcentral gyrus, and paracentral lobule mainly projected causal effects to the fusiform gyri and cerebellar regions. The middle occipital gyrus and right cerebellum_4_5 projected causal effects towards each other. For the inflow ROI-ROI GCA (Figure 4B), the cingulate cortex projected causal effects towards the other ROIs and received causal effects from the superior temporal gyri. Also, the Cingulate cortex, middle frontal gyri, basal ganglia, precentral gyrus, SMA, and the right superior temporal gyrus projected causal effects towards the left cerebellum crus2, which served as a causal target.

## 4 Discussion

This study investigated the structural differences in ADHD and the causal relationship between the right pallidum and whole-brain and among significant outflow and inflow regions using VBM and signed path coefficient GCA. Our analyses revealed the following: (1) structural differences among the three ADHD groups were mainly in the bilateral thalamus, left pallidum, bilateral insula, superior temporal cortex, right cerebellum, and the right pallidum which showed a positive correlation with overall disease severity; (2) the seed region (right pallidum) showed causal effects on the Occipital_Mid_R, bilateral fusiform, Postcentral_L, Paracentral_Lobule_L, Amygdala_L, and the right cerebellum; (3) causal effect from the cerebellum_Crus2_L, Putamen_L, Caudate_L, bilateral superior temporal pole, prefrontal cortex, cingulate cortex, Precentral_R, and the Supp_Motor_Area_L to the seed region were observed; and (4) ROI-ROI GCA demonstrated directional causal relationships among ROIs. To the best of our knowledge, our study is the first to investigate structural differences among the three ADHD age groups, the causal relationship between the right pallidum and whole-brain, and the interregional causal relationships among these ROIs.

### 4.1 Structural differences in ADHD

The significant gray matter volume differences observed in ADHD patients in the current study were found in the bilateral pallidum, bilateral thalamus, bilateral insula, superior temporal cortex, and the right cerebellum. Reduced globus pallidus volume in ADHD patients has been reported in several previous ADHD studies (Frodl and Skokauskas, 2012; Hoogman et al., 2017). The pallidum regulates voluntary movement (Gillies et al., 2019); specifically, hyperkinesia is among the pronounced characteristic symptoms in ADHD patients (Sempere-Tortosa et al., 2021); that is, damage to the pallidum, as shown in this study, implicates movement disorder. Also, the correlation analysis indicating a positive relationship between the right pallidum and disease severity; implied that the higher the reduction of gray matter volume in the right pallidum, the higher the disease severity. Consistent with previous studies (Mills et al., 2012), the thalamus has been found to be implicated in ADHD patients. The thalamus plays several crucial roles, such as regulating alertness and consciousness and relaying sensory and motor signals between the brain and the body (Torrico and Munakomi, 2022). Defects in this region may complicate motor, sensory and cognitive functioning in ADHD patients. The insula cortex, which is part of the salience network, has been linked with symptoms of ADHD and plays an essential role in affective and cognitive processes (Menon and Uddin, 2010; Janes et al., 2018). Consistent with our findings, previous studies have found structural deficits and functional abnormalities in the insula of ADHD patients. Lopez-Larson et al. (2012) reported decreased gray matter volume in the anterior bilateral insula, associated with attention and inhibition problems. Some recent works also showed reduced functional connectivity between the amygdala and insula interaction in regulating emotion in ADHD children (Hulvershorn et al., 2014; Yu et al., 2020); these findings further show the implication of the insula in ADHD. The superior temporal gyrus, one of the gyri of the temporal lobe, has been reported to exhibit reduced activation and overlap with decreased gray matter volumes in the putamen and anterior insula in ADHD patients (Norman et al., 2016). The superior temporal gyrus is involved in language and mediates spatial awareness and exploration (Karnath, 2001); hence alterations in the gray matter volume of this brain region in the current study might imply language and spatial awareness impairments in ADHD patients. Consistent with previous studies, the cerebellum also exhibited defects in gray matter volume in the present study. Pathogenesis of ADHD is associated with abnormalities in the function and morphology of fronto-striatal brain regions (Durston et al., 2011). However, recent studies suggest that structural and functional abnormalities in ADHD are not limited to the fronto-striatal areas but to other regions, including the thalamus (Ivanov et al., 2010; Mills et al., 2012), the cerebellum (Valera et al., 2007), among others. The cerebellum is involved in motor control and higher cognitive processes, including learning (Steinlin, 2008), attention shifting (Golla et al., 2005), visual-spatial processing (Ivry et al., 2002), working memory (Cooper et al., 2012; Stoodley et al., 2012), and emotion (Ferrucci et al., 2012). Accordingly, the deficit of the cerebellum may suggest impairments in cognitive control and attentional mechanisms in ADHD patients.

### 4.2 Effective connectivity

For effective connectivity, the right pallidum as a seed demonstrated causal effects (outflow) on the Occipital_Mid_R, bilateral fusiform, Postcentral_L, Paracentral_Lobule_L, Amygdala_L, and the right cerebellum indicating that the right pallidum precedes and allows prediction of these brain regions. Also, the anterior cingulate cortex, prefrontal cortex, cerebellum_Crus2_L, Putamen_L, Caudate_L, bilateral superior temporal pole, middle cingulate cortex, Precentral_R, and the Supp_Motor_Area_L demonstrated causal effects on the seed region (inflow), indicating that these brain regions precedes and allow prediction of the right pallidum. The ROI-ROI GCA showed a directional network for both outflow and inflow. The outflow ROI directional network demonstrated that the right pallidum was its pivot. Disruptions in the right pallidum might be related to disruptions in other brain areas in the outflow connectivity graph (Figure 4A). The amygdala was identified as the transition point and received causal effects from the right pallidum and projected to other regions. The right fusiform gyri and the cerebellar regions received more causal effects from the other regions and were identified as the causal targets. The ACC was identified as the core of the inflow ROI directional causal relationships (Figure 4B). Changes in the ACC potentially conferred causal effects to all other regions. Consistent with previous ADHD studies (Frodl and Skokauskas, 2012; Bledsoe et al., 2013), the ACC has been reported to be implicated in ADHD. More projections were made from the frontal cortex and basal ganglia regions to the left cerebellum, suggesting frontal-striatal-cerebellar connectivity, reported in ADHD (Giedd et al., 2001). The cerebellum has been identified as a causal target in both inflow and outflow ROI causal relationships; this further provides evidence of the involvement of the cerebellum in ADHD pathophysiology. The GCA captured the effect of the abnormality of the right pallidum on other regions, the effects of the abnormalities in other regions on the right pallidum, and the directional causal relationships among these brain regions.

The occipital cortex is responsible for visual processing and has been reported in previous ADHD studies (Wolf et al., 2009; Sokunbi et al., 2013); therefore, the seed’s prediction of this brain region indicates the implication of the occipital cortex in ADHD. Consistent with previous studies (Hoogman et al., 2019), the fusiform also showed activation in the current study. This activation of the bilateral fusiform was caused by the neural activity in the right pallidum. The occipital cortex and the fusiform gyri are part of the visual network, suggested to be responsible for processing information about static and moving objects, spatial awareness and guidance of action, and object recognition (James et al., 2003; Nassi and Callaway, 2009) and have been reported to show abnormal connectivity in several ADHD studies (Benli et al., 2018; Agoalikum et al., 2021). Our results also showed that the neural activity in the seed region could predict the neural activity in the left postcentral gyrus and the left paracentral lobule, which are part of the somatosensory system reported to be implicated in ADHD (Parush et al., 1997). The somatosensory system is associated with touch, nerve pathways, and parallel receptors for the sensation of pain, temperature, body position, and movement (Sherman, 2019). The somatosensory cortex is also said to be involved in emotional regulation (Kropf et al., 2019), which is suggested to be associated with ADHD (Retz et al., 2012; Corbisiero et al., 2013). The amygdala is said to be involved in emotional regulation especially negative emotions (Bonnet et al., 2015); hence, the activation of the left amygdala caused by the right pallidum might suggest emotional impairment in ADHD patients. Previous structural and functional studies have reported abnormal connectivity in the cerebellum of ADHD patients (Valera et al., 2007; Agoalikum et al., 2021). Deficits in gray matter volume and cerebellum activation further provide evidence of the cerebellum’s implication in ADHD. The cingulate cortex, especially the anterior cingulate cortex (ACC), has been reported to show symptom severity and dysfunction in attention, motor control, and volumetric reductions in ADHD patients (Makris et al., 2007; Bush et al., 2008; Frodl and Skokauskas, 2012; Bledsoe et al., 2013; Sanfratello et al., 2019), hence the ACC predicting the right pallidum in the current study provides further evidence of the implication of this region in ADHD. The prefrontal cortex is essential in cognitive control functioning, thereby regulating attention and behavior via its extensive connectivity to the motor and sensory cortices, as well as subcortical regions like the cerebellum and basal ganglia (Arnsten, 2009). The prefrontal cortex, cerebellum, and the basal ganglia regions predicted the right pallidum (seed) in the current study; this is consistent with the frontal-striatal-cerebellar circuits reported in ADHD (Giedd et al., 2001), suggesting that the prefrontal cortex and its extensive connections are associated with ADHD symptoms. The Right precentral and left supplementary motor areas which are part of the sensorimotor network (SMN), also predicted the seed region in the current study. The SMN is involved in the coordination of sensory and motor actions and has been associated with hyperactivity in ADHD patients (Sörös et al., 2019). Our results also showed that neural activity in the bilateral superior temporal pole could predict the neural activity in the right pallidum, which shows defects in gray matter volume in the current study. The temporal pole is involved in functions such as semantic processing, memory, and emotional and social behavior, among others (Córcoles-Parada et al., 2019), which may be associated with ADHD.

Although we showed the structural differences in ADHD and the causal relationship between the right pallidum and whole brain, as well as among outflow and inflow ROIs in the three ADHD patient groups, the current study has some limitations. Albeit the control of sex variable in the statistical analysis, the male to female ratio in the current study was not the same, which may affect the results. Also, the sample size was a limitation in this study, as connectivity tends to be more stable with an increasing number of participants. We were not able to investigate whether our relatively small sample influenced the result presented, we suggest that future studies recruit larger sample sizes to include a balanced sex ratio.

In conclusion, the current study investigated the structural differences among ADHD patients and the causal relationship between the right pallidum and the rest of the brain and among significant outflow and inflow regions, using VBM and coefficient-based GCA. The significant gray matter volume defects in the right pallidum were positively correlated with disease severity. Also, significant outflow and inflow regions obtained from the GC analysis revealed directional causal relationships among ROIs. Generally, the current study demonstrated structural differences among the three ADHD patient groups and the ability of GCA to capture the causal connectivity between brain regions. Furthermore, this work highlights the evidence of the frontal-striatal-cerebellar circuits in ADHD and provides new insights into the effective connectivity of the right pallidum and the pathophysiology of ADHD.

## Data availability statement

Publicly available datasets were analyzed in this study. These data can be found here: http://fcon_1000.projects.nitrc.org/indi/adhd200/ and https://openneuro.org/datasets/ds000030/.

## Author contributions

HW, PH, and JJ: organized and preprocessed the data. EA and HW: formal analysis. EA and BK-B: statistical analysis and results. EA: writing the first draft. BK-B and BB: revision and editing. All authors contributed to the article and approved the submitted version.

## Footnotes

^1^ https://neuro-jena.github.io/cat/

^2^ http://www.fil.ion.ucl.ac.uk/spm/

^3^ https://neuro-jena.github.io/software.html#tom

^4^ https://marsbar-toolbox.github.io/index.html

^5^ http://www.restfmri.net
