# Supplementary material for: Structural differences among children, adolescents, and adults with attention-deficit/hyperactivity disorder and abnormal Granger causality of the right pallidum and whole-brain
Source: Front Hum Neurosci. 2023 Feb 14;17:1076873. doi: 10.3389/fnhum.2023.1076873 (PMC9971633; doi:10.3389/fnhum.2023.1076873)
Supplement: Supplementary file 1 [file Data_Sheet_1.docx]

Supplementary Material

**Structural Differences Among Children, Adolescents, and Adults With Attention Deficit Hyperactivity Disorder and Abnormal Granger Causality of the Right Pallidum and Whole-Brain.**

^1^ The Clinical Hospital of Chengdu Brain Science Institute, MOE Key Laboratory for Neuroinformation, School of Life Science and Technology, University of Electronic Science and Technology of China, No.2006, Xiyuan Avenue, West Hi-Tech Zone, Chengdu, Sichuan 611731, China

^2^ Department of Biomedical Engineering, New Jersey Institute of Technology, 619 Fenster Hall, Newark, NJ 07102, USA

**Data availability statement**

The data is available from the corresponding authors upon reasonable request.

**Funding statement**

This work was supported by the National Natural Science Foundation of China (NSFC61871420, NSFC62171101).

**Conflict of interest disclosure**

The authors report no conflicts of interest.

*** Correspondance**Bharat Biswal
[bbiswal@gmail.com](mailto:bbiswal@gmail.com)

Benjamin Klugah-Brown

[bklugah@gmail.com](mailto:bklugah@gmail.com)

**Table S1:** Regions showing reduced grey matter volume among the three ADHD age groups.

| Brain Region | MNI Coordinates (X Y Z) | Peak Intensity | Cluster size (Voxels) |
| --- | --- | --- | --- |
| Pallidum_R | 18 2 -2 | 17.9533 | 203 |
| Pallidum_L | -17 0 -2 | 19.0393 | 317 |
| Thalamus_R | 18 -27 11 | 14.0834 | 412 |
| Thalamus_L | -15 -32 9 | 21.256 | 2718 |
| Insula_R | 23 23 -8 | 8.0936 | 39 |
| Insula_L | -33 17 -11 | 13.0992 | 183 |
| Temporal_Sup_L | -51 6 -5 | 15.9772 | 4074 |
| Cerebelum_8_R | 30 -65 -51 | 11.2151 | 526 |

Table S2: Regions showing causal effect from the right pallidum to whole-brain.

| Brain Region | MNI Coordinates (X Y Z) | Peak Intensity | Cluster size (Voxels) |
| --- | --- | --- | --- |
| Fusform_R | 25 -71 -6 | 31.47391 | 95 |
| Fusiform_L | -22 -39 -14 | 31.42960 | 103 |
| Paracentral_Lobule_L | -1 -31 58 | 18.50077 | 50 |
| Postcentral_L | -35 -31 50 | 27.63033 | 110 |
| Occiptal_Mid_R | 36 -86 4 | 40.32933 | 112 |
| Cerebelum_4_5_R | 22 -35 -20 | 20.08122 | 68 |
| Cerebelum_6_R | 27 -54 -19 | 19.98738 | 87 |
| Amygdala_L | -21 -4 -17 | 21.82373 | 81 |

Table S3: Regions showing causal effect from Whole-brain to the right pallidum

| Brain Region | MNI Coordinates (X Y Z) | Peak Intensity | Cluster size (Voxels) |
| --- | --- | --- | --- |
| Cerebelum_Crus2_L | -3 -81 -36 | 14.8415 | 141 |
| Cingulum_Mid_L | -2 26 35 | 18.40725 | 83 |
| Caudate_L | -11 13 0 | 11.78759 | 91 |
| Temporal_Pole_Sup_R | 44 -1 -16 | 11.44691 | 104 |
| Cingulum_Ant_L | -3 39 0 | 21.9265 | 660 |
| Putamen_L | -18 15 0 | 16.7191 | 46 |
| Temporal_Pole_Sup_L | -54 9 0 | 15.7154 | 42 |
| Frontal_Mid_R | 48 45 24 | 21.7851 | 371 |
| Frontal_Mid_L | -42 23 50 | 10.71502 | 88 |
| Precentral_R | 64 10 23 | 11.24509 | 93 |
| Supp_Motor_Area_L | -4 12 50 | 12.25850 | 95 |
